# Tracing CLL-biased stereotyped immunoglobulin gene rearrangements in normal B cell subsets using a high-throughput immunogenetic approach

**DOI:** 10.1186/s10020-020-00151-9

**Published:** 2020-03-10

**Authors:** Monica Colombo, Davide Bagnara, Daniele Reverberi, Serena Matis, Martina Cardillo, Rosanna Massara, Luca Mastracci, Jean Louis Ravetti, Luca Agnelli, Antonino Neri, Michela Mazzocco, Margherita Squillario, Andrea Nicola Mazzarello, Giovanna Cutrona, Andreas Agathangelidis, Kostas Stamatopoulos, Manlio Ferrarini, Franco Fais

**Affiliations:** 1 U.O. Molecular Pathology, IRCCS Ospedale Policlinico San Martino, Genoa, Italy; 2 Department of Experimental Medicine, University of Genoa, Genoa, Italy; 3 U.O. Pathology, IRCCS Ospedale Policlinico San Martino, Genoa, Italy; 4 Department of Surgical Sciences and Integrated Diagnostics, University of Genoa, Genoa, Italy; 5 Department of Oncology and Hemato-oncology, University of Milan, Milan, Italy; 6 U.O. Laboratorio di Istocompatibilità, E.O. Ospedali Galliera, Genoa, Italy; 7 Department of Informatic Bioengeneering, Robotic and System Engeneering, University of Genoa, Genoa, Italy; 8 The Feinstein Institute for Medical Research, Manhasset, NY USA; 9 Institute of Applied Biosciences, Center for Research and Technology Hellas CERTH, Thessaloniki, Greece

## Abstract

**Background:**

B cell receptor Immunoglobulin (BcR IG) repertoire of Chronic Lymphocytic Leukemia (CLL) is characterized by the expression of quasi-identical BcR IG. These are observed in approximately 30% of patients, defined as stereotyped receptors and subdivided into subsets based on specific VH CDR3 aa motifs and phylogenetically related IGHV genes. Although relevant to CLL ontogeny, the distribution of CLL-biased stereotyped immunoglobulin rearrangements (CBS-IG) in normal B cells has not been so far specifically addressed using modern sequencing technologies. Here, we have investigated the presence of CBS-IG in splenic B cell subpopulations (s-BCS) and in CD5^+^ and CD5^−^ B cells from the spleen and peripheral blood (PB).

**Methods:**

Fractionation of splenic B cells into 9 different B cell subsets and that of spleen and PB into CD5^+^ and CD5^−^ cells were carried out by FACS sorting. cDNA sequences of BcR IG gene rearrangements were obtained by NGS. Identification of amino acidic motifs typical of CLL stereotyped subsets was carried out on IGHV1-carrying gene sequences and statistical evaluation has been subsequently performed to assess stereotypes distribution.

**Results:**

CBS-IG represented the 0.26% average of IGHV1 genes expressing sequences, were detected in all of the BCS investigated. CBS-IG were more abundant in splenic and circulating CD5^+^ B (0.57%) cells compared to CD5^−^ B cells (0.17%). In all instances, most CBS IG did not exhibit somatic hypermutation similar to CLL stereotyped receptors. However, compared to CLL, they exhibited a different CLL subset distribution and a broader utilization of the genes of the IGHV1 family.

**Conclusions:**

CBS-IG receptors appear to represent a part of the “public” BcR repertoire in normal B cells. This repertoire is observed in all BCS excluding the hypothesis that CLL stereotyped BcR accumulate in a specific B cell subset, potentially capable of originating a leukemic clone. The different relative representation of CBS-IG in normal B cell subgroups suggests the requirement for additional selective processes before a full transformation into CLL is achieved.

## Introduction

Chronic lymphocytic leukemia (CLL) is characterized by pronounced clinical heterogeneity that likely reflects the underlying biological complexity (Chiorazzi and Ferrarini 2011; Zenz et al. 2010). A fundamental aspect of CLL ontogeny and evolution concerns microenvironmental interactions mediated through various surface receptors, amongst which the B cell receptor immunoglobulin (BcR IG) plays perhaps the most prominent role. This is amply demonstrated by the segregation of CLL cases into two subgroups with widely divergent clinical courses based on the impact of the somatic hypermutation (SHM) burden within the clonotypic rearranged immunoglobulin heavy variable (IGHV) gene. One subgroup exhibits high germline identity (GI ≥ 98%, Unmutated or UM-CLL) and is generally associated with adverse prognosis, whereas the other subgroup is characterized by a substantial SHM load (GI < 98%, Mutated or M-CLL) and generally presents with an indolent disease course (Damle et al. 1999; Hamblin et al. 1999).

Over the years, several independent studies have shown that CLL displays a skewed IGHV gene repertoire, largely different from that of normal circulating B cells (Schroeder Jr. and Dighiero 1994; Fais et al. 1998). Moreover, despite the enormous potential of BcR IG diversity, up to 30% of CLL patients carry quasi-identical BcR IG. Such highly restricted BcR IG were defined as stereotyped (Messmer et al. 2004; Widhopf et al. 2004) and patients carrying such rearrangements were classified accordingly into distinct subsets, each defined by a unique sequence amino acid (aa) motif within the heavy variable complementarity-determining region 3 (VH CDR3) (Agathangelidis et al. 2012; Agathangelidis et al. 2014). Although several hundred subsets have been reported to date, 19 of these were larger in size, cumulatively accounted for more than 10% of BcR of all CLL clones, and were thus defined as major subsets (Agathangelidis et al. 2012). The assignment of a given patient to a stereotyped subset requires particular BcR IG features including the aa length and composition of the VH CDR3 along with the usage of a certain IGHV gene. Specifical rules determine the degree of VH CDR3s diversity: the assignment to the same stereotyped subset implies at least 50% aa identity and 70% aa similarity as well as identical VH CDR3 length and offset of the sequence pattern (Agathangelidis et al. 2012; Darzentas and Stamatopoulos 2013). Moreover, these VH CDR3 have to be associated with IGHV genes belonging to the same phylogenetic clan, i.e. genes that are evolutionarily related, hence quite similar at the sequence level (Le Franc Lefranc M-PaL, G 2001; Kirkham et al. 1992).

These remarkable features of the BcR IG gene repertoire led to the notion that antigenic stimulation is a critical factor in the natural history of CLL (Chiorazzi et al. 2005; Ghia and Caligaris-Cappio 2006). Several studies have demonstrated that the outcome of antigen engagement results in a down-modulation of sIgM in CLL cells that appears to be less pronounced in the UM-CLL compared to M-CLL (Stevenson et al. 2011; Coelho et al. 2013; Apollonio et al. 2013; Drennan et al. 2019). In addition, it has been reported the possibility that homotypic interactions between the BcR IG present on the clonal cell surface, leading to receptor de-clustering allow the activation of a cell autonomous signaling cascade that may ultimately induce cell activation/proliferation (Duhren-von Minden et al. 2012; Minici et al. 2017).

Stereotyped BcR IG have also been identified in other mature B cell malignancies (Agathangelidis et al. 2019) as well as in healthy individuals (Forconi et al. 2010; Seifert et al. 2012). In more detail, stereotyped BcR IG were evident in mantle cell lymphoma (Hadzidimitriou et al. 2011) and at lower frequencies in splenic marginal zone lymphoma (Zibellini et al. 2010; Bikos et al. 2012). Furthermore, “CLL-biased” BcR IG were found in monoclonal B cell lymphocytosis, a known pre-CLL condition (Morabito et al. 2013; Vardi et al. 2013; Agathangelidis et al. 2017). However, very few studies have investigated the existence of CLL-like stereotypes in settings other than CLL with high throughput sequencing techniques (Vergani et al. 2016) and no one in spleen B cells.

In this study, we used next generation sequencing (NGS) to collect highly resolved information on the presence of CLL-biased stereotyped BcR IG in splenic normal B cell subpopulations (s-BCS). In addition, CD5^+^ and CD5^−^ B cells were isolated and analyzed either from spleen or from peripheral blood (PB). We focused on IGHV-IGHD-IGHJ gene rearrangements utilizing IGHV1 genes, namely the subgroup where falls 9 of the 19 major CLL stereotyped subsets (Agathangelidis et al. 2012; Darzentas et al. 2010).

## Materials and methods

### Sample origin

Six spleens, free of neoplastic cells at histological inspection, were obtained from the Pathology Division of the San Martino Hospital. Spleen 19 (from a patient aged 87), spleen 25 (p. aged 81), spleen 26 (p. aged 21), spleen 32 (p. aged 31) and spleen 41 (p.aged 73) were obtained from patients who had been splenectomized following trauma. Spleen 713 was obtained from a patient aged 78 who had been splenectomized at surgery for a gastric adenocarcinoma (with no spleen involvement).

PB samples were obtained from the Department of Experimental Medicine (University of Genoa) from three normal donors aged 54 (WS), 66 (EC) and 75 (AZ), respectively. This age range was selected to approximately match the age of CLL onset.

### Isolation of splenic B cell subpopulations and fractionation of CD5^+^ and CD5^−^ B cells

Spleen cell suspensions were obtained using a gentle Macs dissociator (Miltenyi Biotech GmbH, Bergisch Gladbach, Germany) and mononuclear cells were separated by density gradient separation using Ficoll Hypaque (Seromed; Biochrom KG, Berlin, Germany).

B cell subpopulations were isolated by staining with mAbs tagged with different fluorochromes followed by sorting on a two laser (488 nm and 640 nm) FACS (FACSAria, Becton Dickinson Italia, Milano, Italy), as detailed below and depicted in Supplementary Fig. 1 and Supplementary Table 1. The following combination of mAbs was employed: anti CD19 APC-Vio770 (Miltenyi Biotech); anti-IgD Alexa Fluor 488 (BioLegend, San Diego, CA); anti-IgM PE, anti-CD27 PE-CF594, anti-CD38 PE-Cy7, anti-CD24 Alexa Fluor 647 and anti-CD5 Alexa Fluor 700 (BD).

Operationally, splenic CD19^+^ B cells were first fractionated according to the expression of CD38 and IgD (Supplementary Fig. 1A, top line, center (Colombo et al. 2013). The following B cell subpopulations were then isolated: follicular mantle (FM) B cells were isolated as IgD^++^, CD38^−^ B cells and depleted further of CD27^+^ cells (top line, right). Germinal center (GC) B cells were isolated as CD38^+^, IgD^−^ B cells and depleted further of CD24^+^ cells (top line, left). Switched-memory (SM) B cells were identified as IgD^−^CD38^−^IgM^−^ (middle line, center), while MZ B cells were separated as IgD^low-^CD38^neg^IgM^+^ cells (middle line, center). Besides, in spleens 19, 32 and 41, the IgM^+^ cell fraction obtained from the CD38^−^, IgD^low/neg^ cells (middle line, center) was separated further into IgM only (MO) B cells (IgD negative) and MZ B cells (middle line, center) that were further fractionated into CD27^+^ (MZ27^+^) and CD27^−^ (MZ27^−^) B cells (middle line, right). Moreover, SM B cells were further depleted of CD27 to obtain double-negative (DN) B cells (middle line, left). Transitional (TR) B cells were obtained by gating CD19^+^ B cells expressing CD38^++^ and CD24^++^ (Supplementary Fig. 1B).

One of the spleens (SPL25) was studied only for the main B cell subpopulations (FM, GC, MZ and SM).

Only IgM transcripts were analyzed in the majority of the B cell subpopulation samples indicated above; both IgM and IgG transcripts were analyzed in GC B cell samples. Only IgG transcripts were analyzed in the case of SM and DN B cell samples.

Cells were sorted into 200 μl PCR tubes containing 100 μl (Ambion-Thermofisher Scientific, Waltham, MA. USA) binding lysis buffer and stored at − 20 °C.

Technical replicates were taken by isolating aliquots of the same B cell subpopulation samples in different tubes: they were processed and run separately, then analyzed together for the clonal assignment in the bioinformatics pipeline.

In order to separate CD5^+^ and CD5^-^ cells from the splenic and peripheral blood cell suspensions, CD19- positive B cells were gated in the FACS-sorter and subsequently fractionated into CD5-positive and CD5-negative cells (Drennan et al. 2019; Darzentas et al. 2010; Seifert and Kuppers 2016).

### Library preparation and sequencing

High-throughput sequencing analysis of IGHV-IGHD-IGHJ gene rearrangements was performed on total RNA as previously described (Vergani et al. 2017). Briefly, mRNA was isolated using poly-T (Ambion, Thermofisher) coupled with magnetic beads (Supplementary Fig. 2). Total mRNA was reverse transcribed in this solid phase by using the poly-T as primers for the reaction. Beads bound to cDNA were then purified with a magnet and cDNA was used to synthetize ds-cDNA of the IGHV-IGHD-IGHJ gene rearrangements. The original protocol (Vergani et al. 2017) was partially modified in spleens 713, 26 and 25 and limited to IGHV1 subgroup genes sequencing. To this end, six different primers that anneal to the 5′ end of the leader sequence of all IGHV1 subgroup genes were multiplexed in a single PCR (supplementary M&M). PCR was performed in solid phase in 10 μl (polymerase activation: 98 °C for 30 s, denaturation: 98 °C for 15 s, annealing: 60 °C for 5 min and elongation including final extension of: 72 °C for 5 min), using a Q5 DNA High Fidelity DNA Polymerase (NEB, Ipswich, MA, USA). Primers were composed of 14 to 16 random nucleotide sequences at the 5′ end and partial Illumina Adaptor sequences as detailed in supplementary M&M.

Double-stranded cDNA was used as template for a second PCR amplification round using a Q5 High Fidelity DNA Polymerase, a universal forward primer (ILF1) and reverse isotype specific primers mu and gamma constant region that contained Illumina Adaptor sequences (supplementary M&M). Only IGHV1 subgroup gene sequences associated with mu and gamma specific primers sequences were analyzed in this study.

CD5^+^ and CD5^−^ B cells isolated from PBL and were analyzed separately from the other s-BCS.

PB CD5^+^ and CD5^−^ cells and all cells from spleen 19, 32 and 41 were amplified according to the Vergani protocol (Vergani et al. 2017) by using IGHV primers for all the IGHV subgroups in conjunction with primers specific for mu, gamma, delta and alfa constant regions. Only IGHV1 subgroup gene sequences associated with mu and gamma specific primer were analyzed in this study.

PCR conditions were the following: 98 °C for 30 s (polymerase activation); 22 cycles of: 98 °C for 15 s (denaturation), 60 °C for 30 s (annealing), 72 °C for 30 s (extension); 72 °C for 7 min (final extension). The PCR product was purified by using Ampure XP beads (Beckman Coulter, Brea, CA, USA) at ratio of 1:0.7x; 1 μl of PCR product was run on TapeStation (Agilent Technologies, Santa Clara, CA, USA) by using the High sensitivity D1000 Screen Tape to control amplicon quality. Products were quantified with Qubit 2.0 (Thermofisher). One ng was used to add Illumina Index Primers with the Nextera XT kit (Illumina, San Diego, CA, USA). A 2 × 300 bp paired end kit (MiSeq V3, 2 × 300 kit, Illumina) was used for library generation and sequencing. The library was loaded at 12 pM with 5% PhiX. DNA sequences were deposited on Sequence Read Achive (SRA) portal of NCBI with BioProject ID: PRJNA515424.

### Bioinformatics analysis

In order to ensure consistency, all sequences were subjected to a multi-level process of filtering. Raw NGS reads were first processed with a custom-built workflow using pRESTO (Vander Heiden et al. 2014) as detailed in Supplementary M&M (Code 1). Selected IGHV1 subgroup gene rearrangement sequences in association with gamma or mu constant heavy chain were submitted to the IMGT/HighV-QUEST tool (Alamyar et al. 2012).

Curated sequences were then scanned for the presence of aa motifs within the VH CDR3 that are characteristic for the major CLL stereotyped subsets with the application of a previously described algorithm (Agathangelidis et al. 2012).

After this analysis, sequences were clustered into two distinct groups: either CLL-biased stereotyped (CBS-IG) and non-CBS-IG (i.e. not assigned to CLL stereotyped subsets), and then categorized into clonal families by using ChangeO (Gupta et al. 2015). Briefly two IGHV-IGHD-IGHJ gene rearrangement sequences were assigned to the same clonal family if they were using the same IGHV, and IGHJ genes, presenting a VH CDR3 with the same length and a maximum VH CDR3 nucleotide distance (threshold) calculated by findThreshold function from the SHazaM R package. Threshold values are comprised from 0 to 1 (0 = 100% identity, 1 = 0% identity). The optimal threshold was calculated for each individual sample and s-BCS (supplementary Table 2). Code commands are reported in supplementary MM (Code 2). Briefly, recurrent sequences assigned to the same clonal group were collapsed to a single IGHV-IGHD-IGHJ gene rearrangement representative of each clonal family. Figures and statistical analyses have been made at clonal level, i.e. considering only “one representative sequence” from each clonal family. Conventionally, the representative IGHV-IGHD-IGHJ rearrangement was the sequence with the highest number of sequenced mRNA molecules sequenced. Data of s-BCS and CD5^+^ and CD5^−^ B cells are summarized in supplementary Table 2.

### Statistics

Statistical analyses were performed in R. Contingency tables were analyzed using Fisher’s exact test. Kruskal-Wallis and Dunn’s tests with Bonferroni correction and Cochrane Mantel Haenszel test were applied to assess differences in CBS-IG frequency and in group distributions, respectively (significance at *p* < 0.05 in all tests).

## Results

### Processing of sequence data and identification of CLL-biased stereotyped immunoglobulin gene rearrangements (CBS-IG) of the splenic B cell subpopulations (s-BCS)

A total of 182,783 productive, unique IGHV1 subgroup gene rearrangement curated sequences were obtained from 1,315,000 sorted B cells (~ 18%) from six spleens separated as detailed in Supplementary Fig. 1. Curated sequences were subsequently clustered into 117,027 clonal families (detailed in Supplementary Table 2). Two hundred and seventy-nine IGHV-IGHD-IGHJ gene rearrangements were assigned to one of the major IGHV1 CLL stereotyped subsets corresponding to 0.24% of all identified clonal families.

### Frequency of the CLL stereotyped subsets among the CBS-IG was different compared to CLL clones

Stereotyped BcR from CLL patients are categorized in different subsets(9). For the sake of clarity, the features of CLL subsets using IGHV1 genes are summarized in supplementary Table 3.

Most CBS-IG clones were assigned to subset #12 (142 clones, 0.12% of the total clones), followed by subset #28A (57 clones, 0.048%). Subsets #7H (21 clones, 0.018%), #3 (23 clones, 0.019%) and #5 (17 clones, 0.014%) were represented at a similar level. Subsets #6 and subset #1–99 were less represented with 10 (0.009%) and 6 (0.005%) clones, respectively. Finally, only 3 clones were detected for subset #59.

**Fig. 1:**
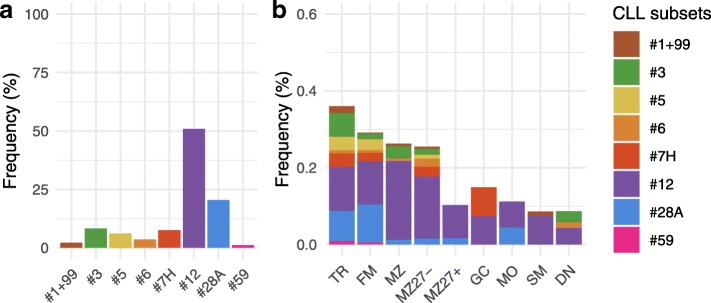
Relative frequency of different stereotype subsets among the CBS-IG rearrangements of s-BCS. **a** Frequency of subsets used by CBS-IG rearrangements of B cells considered in bulk. **b** Relative frequency of the subsets found among the CBS-IG rearrangements expressed by the various s-BCS. Stereotype subsets are marked by different colours as indicated

The relative frequency of the different CLL major stereotyped subsets among CBS-IG rearrangements is shown in Fig. 1a. This distribution of CBS-IG rearrangements among individual major stereotyped subsets was compared to that of CLL reported in the study by *Agathangelidis* et al. *(*Agathangelidis et al. 2012*)*. Because of the study design, the comparison was restricted to CLL sequences utilizing IGHV1 subgroup genes. The frequency of stereotyped subsets in both the CBS-IG and the CLL sequences is depicted in Supplementary Fig. 3. Subset distribution was significantly different (*p* = 0.00049) between the two groups. For example, stereotyped subset #1, which is the most frequent IGHV1 subset in CLL, was scarcely represented in the CBS-IG rearrangements with just 10 clones detected. In contrast, CBS-IG belonging to subsets #12, #28 and #7H, which were the most abundant amongst CBS-IG rearrangements, are much less frequent in the CLL dataset.

### CBS-IG were identified in most of s-BCS

The distribution of stereotyped CBS-IG among different s-BCS showed that such rearrangements were found in all studied s-BCS (Fig. 1b). An increased frequency was observed in TR, FM, MZ and MZ27^−^ B cells yet statistical significance was not reached (Fig. 1b).

Subset #12 CBS-IG sequences were the most represented and this held when B cells were both considered in bulk or subdivided into different s-BCS (see Fig. 1). The second most used CBS-IG was subset #28A. Detailed data on the representation of CBS-IG in the different s-BCS are reported in Supplementary Table 2 and Supplementary Table 4.

### IGHV1 gene usage was broader in CBS-IG compared to CLL major subsets

**Fig. 2:**
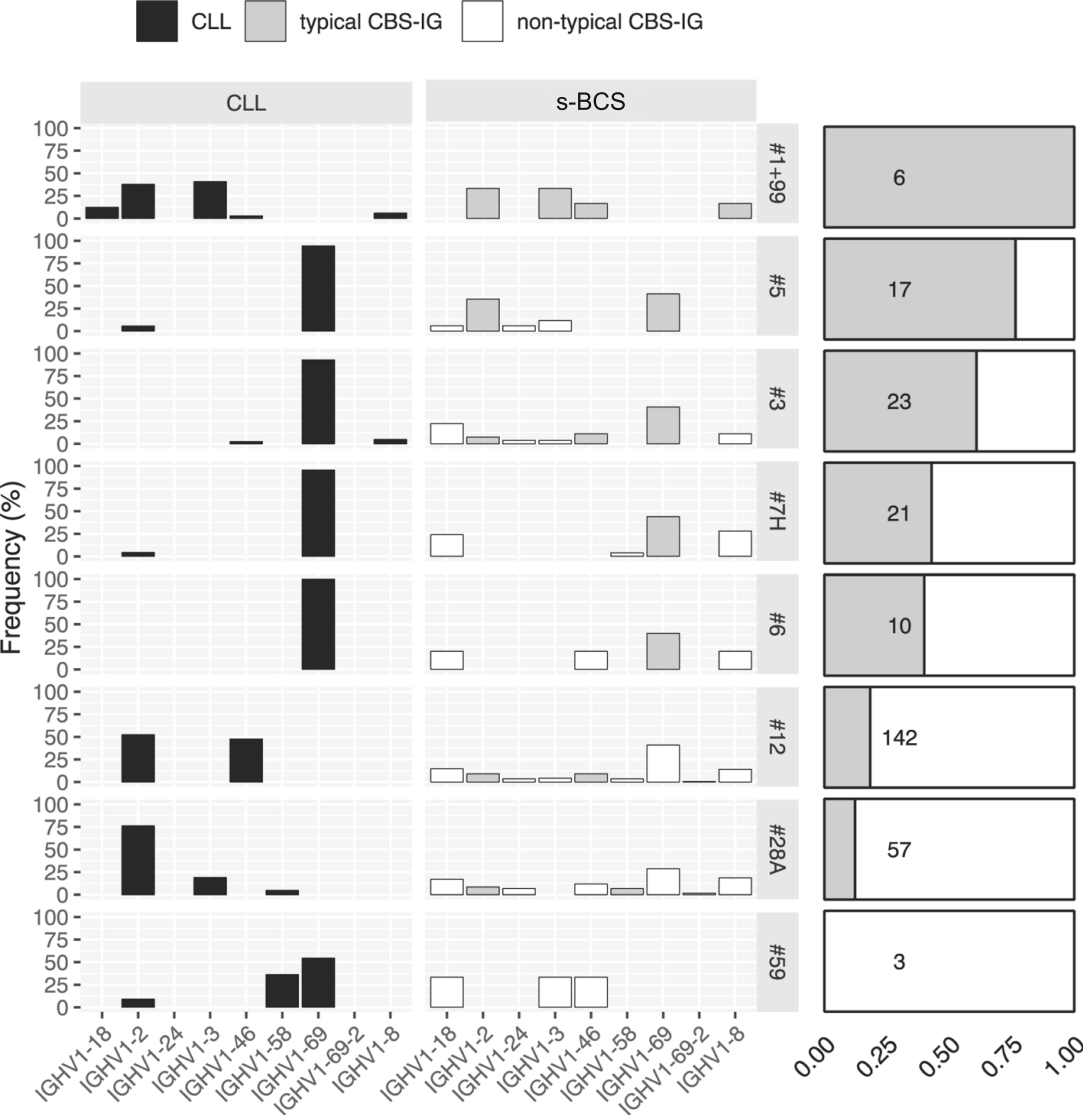
Utilization of IGHV1 family genes by CBS-IG and by CLL stereotype rearrangements. Normal (s-BCS) CBS-IG rearrangements are classified as typical (grey) or non-typical (white) based upon whether the IGHV1 genes utilized were the same or different from those commonly used by CLL clones for a given rearrangement (black). The relative frequency of typical and non-typical CBS-IG in s-BCS (considered in bulk) for each stereotype subset is reported on the right of the figure. Numbers in boxes indicate the total number of sequences investigated for each subset

Next, we compared the IGHV1 gene usage of the CBS-IG with that of CLL clones belonging to the same major stereotyped subsets. The CBS-IG rearrangements expressing the same IGHV1 genes as those usually found in their respective CLL counterparts were here defined as “typical” CBS-IG rearrangements, whereas those expressing a different IGHV1 gene were termed “non-typical”. Both typical and non–typical stereotyped CBS-IG rearrangements were identified in all s-BCS (Supplementary Fig. 4A) revealing an overall broader usage of IGHV1 subgroup genes by CBS-IG rearrangements than CLL (Fig. 2). Stereotyped subsets #1–99, #3, #5, #6 and #7H showed the highest fraction of typical CBS-IG. This was mainly related to the preferential use of the IGHV1–69 gene by all these subsets except subset #1–99 (Fig. 2). In contrast, CBS-IG belonging to subsets #12 and #28A, used IGHV1 subgroup genes different from those frequently found in CLL, with typical rearrangements accounting only for only 18% (26/142) and 12% (7/57) for subsets #12 and #28A, respectively (Fig. 2 and Supplementary Fig. 4B). None of the rare CBS-IG assigned to subset #59 were typical. Individual stereotyped subsets within typical CBS-IG [80/279 = 28.67%] displayed different distribution in comparison with non-typical CBS-IG rearrangements (*p* = 0.00049; Fisher’s test with Monte Carlo simulated *p* value; supplementary Fig. 4B).

### CBS-IG were mostly unmutated

**Fig. 3:**
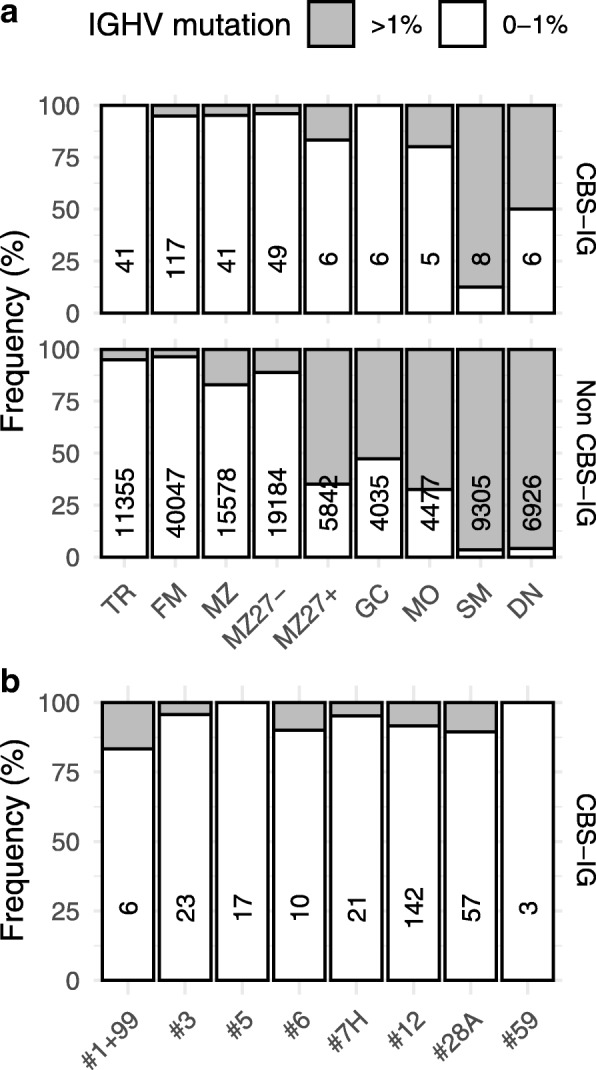
SHM in CBS-IG rearrangements of s-BCS. **a**. Frequency of mutated and unmutated rearrangements in CBS-IG and in non-stereotyped rearrangements (non CBS-IG) in the different s-BCS as indicated. **b** Frequency of mutated and unmutated CBS-IG rearrangements stratified according to CLL stereotype subsets. Numbers in the boxes indicate the total number of clones analyzed

The mutational status of the CBS-IG was determined using a 1% cut-off difference from the corresponding germ line sequence. The majority of the CBS-IG rearrangements from all s-BCS were unmutated (257/279 or 92.1%) (Fig. 3a and b). Exceptions were: (i) SM B cells, which included mostly mutated sequences 7/8 (87.5%) and (ii) DN B cells, that included a fraction of mutated sequences (3/6 or 50%) (Fig. 3a).

In the CLL dataset used in the comparison, these subsets comprised only umutated sequences (except one case belonging to subset #12) with an IGHV germline identity above 99% (or less than 1% difference). This finding makes the comparison between CLL rearrangements and those of s-BCS CBS-IG perfectly feasible even in consideration of the different cut-off set for distinguishing mutated from unmutated sequences in CBS-IG (1%) and CLL rearrangements (2%).

### Identification of CBS-IG in CD5^+^ and CD5^−^ B cells from peripheral blood and spleen

**Fig. 4:**
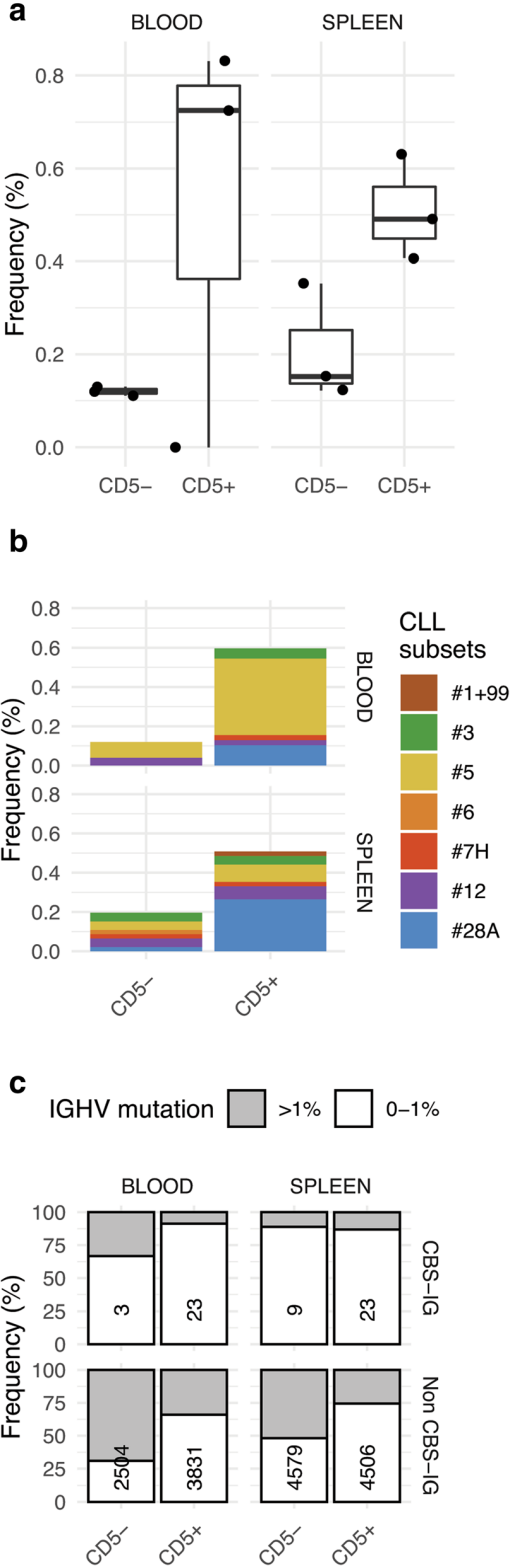
**a** Box-plots of the frequency (%) of CSB-IG rearrangements in CD5^+^ and CD5^-^ B cell fractions from three PB and from three spleen samples. Each dot represents a single individual. CD5^+^ B cells expressed higher number of CBS-IG rearrangements (*p* = 0.002 PB; *p* = 0.01 spleens) irrespective of their location, PB or spleen. **b** CLL stereotyped subset distribution in the CBS-IG rearrangements of CD5^+^ and CD5^-^B cells. CLL stereotype subsets are marked by different colours as indicated. **c** Frequency of mutated and unmutated rearrangements in CBS-IG and in non CBS-IG rearrangements in the CD5^+^ and CD5^-^B cells as indicated

We investigated the representation of CBS-IG in CD5^+^ and CD5^−^ B cells from PB of three subjects and from three spleens. Overall, we analyzed 14,978 productive, unique IGHV1 clones derived from 260,000 cells (details are reported in Supplementary Table 2). The overall representation of CBS-IG was similar in the spleen and in the PB derived B lymphocytes (0.46 and 0.61% respectively). IGHV1 CBS-IG were more frequent in the CD5^+^ cells than in the CD5^-^cells (see Fig. 4a; *p* = 0.01 for the spleen and *p* = 0.002, for the PB). Figure 4b shows the frequency of the different CLL stereotyped subsets among the CBS-IG of the CD5^+^ and CD5^−^ B cells. Subset #5 was predominant in PB CD5+ B cells and subset #28A was the most represented in splenic CD5^+^ B cells, whereas subset #1–99 was scarcely represented in all samples studied (a single clone was identified in the spleen CD5^+^ B cells). Most of CBS-IG gene rearrangements of both CD5^+^ and CD5^-^ B cells were unmutated, while mutated and unmutated sequences were observed in the non CBS-IG gene rearrangements from both CD5^+^ and CD5^-^ cells (Fig. 4c).

## Discussion

The human spleen represents a useful source of s-BCS localized in defined anatomical areas (germinal center, mantle and marginal zone) that can be isolated based on their surface markers (Colombo et al. 2013; Sanz et al. 2008; Weller et al. 2004). Additional markers such as CD24, CD27 and sIg (IgM, IgD IgG and IgA) allow the identification of transitional, memory and naïve compartments. Purified s-BCS enable more detailed BcR IG repertoire analyses than unfractionated B cells, especially for less represented populations that can escape characterization when unfractionated B cells are analyzed. Furthermore, the splenic tissue allows for the isolation of s-BCS that are normally not present in the blood circulation (GC B cells) or, when present (MZ B cells), represent only a small fraction of the subpopulation itself (Bagnara et al. 2015; Seifert and Kuppers 2016; Hendricks et al. 2018). The spleen provides evident advantages for normal, adult BcR IG repertoire analyses over other secondary lymphoid tissues, such as the tonsils, which are frequently removed at young age and in the case of chronic infection. With these premises, we investigated up to nine s-BCS from six different human spleens for the presence of CBS-IG sequences. In addition, CD5^+^ B cells, isolated from spleen and peripheral blood, were investigated also in comparison with the CD5^-^ B cells from the same tissues. These studies were carried out based on the hypothesis that CLL cells may have origin from normal CD5^+^ B cells (Seifert et al. 2012).

CBS-IG rearrangements belonging to subset #12 were the most represented in almost all splenic s-BCS. Sequences belonging to stereotyped subset #12 carry a relatively long VH CDR3, composed of 19 amino acids with a mainly combinatorial amino acidic sequence pattern, i.e. generated primarily through the recombination of the IGHV1/IGHD3–22/IGHJ6 genes with a minor contribution from the N-diversity regions within the junctions (Agathangelidis et al. 2012). This finding could indicate a higher chance of such rearrangements to occur since they require solely a gene combination process. However, other IGHV1-expressing stereotyped subsets equally characterized by combinatorial patterns and by shorter VH CDR3 (i.e. subsets #1, #59 and #99) were less commonly identified. Thus, the size representation of CBS-IG does not appear to merely reflect the likelihood of recombination of the IGHV-IGHD-IGHJ genes. Furthermore, a small number of CBS-IG sequences were mutated and/or isotype switched (IgG) (Supplementary Table 4), indicating that normal B cells carrying stereotyped BcR IG rearrangements could go through all checkpoints during B cell differentiation.

CBS-IG displayed a broader usage of IGHV1 genes than CLL clones and utilized most of IGHV1 subgroup genes. Of interest, the most frequently identified CBS-IG subsets (#12 and #28A) were also those that exhibited an inferior representation of typical CBS-IG (see Fig. 2 and supplementary Fig. 4A). Conversely, in the less frequent CBS-IG subset #1–99 only typical rearrangements were identified (see Fig. 2 and Supplementary Fig. 4A). Thus, these observations as well contribute to establishing differences between the normal and CLL stereotype repertoire.

To further assess the issue of B-cell compartment that represent possible sources of CLL cells, we applied the same techniques (NGS technology and the present bioinformatics pipelines) to study CD5^+^ and CD5^−^ B cells derived from PB and spleen tissues for the presence of CBS-IG rearrangements.

Overall, the data obtained indicated that CD5^+^ B cells are enriched in CBS-IG compared to the CD5^−^ B cells both in spleen and PB tissues (see Fig. 4a and b). As for the splenic CD5^+^ B cells, the relative representation of CBS-IG CLL subsets reflected that observed in the BCS separated in the matched samples (Supplementary Fig. 5). As for CD5^+^ B cells isolated from the PB samples a more frequent representation of CBS-IG belonging to subset #5 was observed (see Fig. 4b and Supplementary Fig. 5). Similarly to the s-BCS described above, subset #1–99 was not observed in PB CD5^+^ B cells and was rarely identified in samples of spleen CD5^+^ B cells (Fig. 4b and Supplementary Fig. 5). Likewise, subset #59 was never observed among CD5^+^ CBS-IG. Thus, CD5^+^ B cells do not appear to have a CBS-IG repertoire reminiscent of that observed in CLL clones (Supplementary Fig. 5B and C).

Regarding CD5^+^ B cells, it is still matter of debate if this marker can identify a truly separate lineage as it happens in mice [reviewed in (Prieto and Felippe 2017)]. The frequency of CBS-IG identified in this study is markedly lower if compared to what reported in the study by *Seifert* et al (Seifert et al. 2012). This might be due to the different number of sequences analyzed, the more restricted set of CLL subsets investigated and mostly, to the criteria used to define BCR stereotypy that are more stringent in the present study.

CD5^+^ B cells isolated from the spleen do not seem to carry significantly more CBS-IG when compared to the other BCS isolated from the same sample (see Supplementary Fig. 5A and B). Related to this, CD5^+^ B cells are represented at various ratios in the s-BCS studied [2–8% of CD19^+^ B cells in spleens vs 24–30% observed in PB (not shown)] with an elevated variation among the samples analyzed. In addition, CD5^+^ (and CD5^−^) B cells isolated for Ig rearrangement sequencing are only partially represented in the other separated s-BCS as a significant proportion of B cells are excluded in the separations operated using the various markers (as shown in Supplementary Fig. 6). Thus, it is not possible to identify a single s-BCS that exclusively (or mostly) harbors CD5^+^ B cells. Further studies are needed to get better insight into this issue.

Some degree of heterogeneity of CBS-IG gene rearrangement representation was observed in the samples from different subjects. However, in none of the samples analyzed, similarity of repertoire with that of CLL patients was observed (Supplementary Fig. 5B and C).

Overall, most CBS-IG sequences did not show a heavy SHM load, in analogy to that found for IGHV1-expressing CLL major stereotyped subsets and indeed these were mostly identified in the CBS-IG unmutated rearrangements (Figs. 3 and 4c).

Based on the above repertoire comparisons, it can be argued that CLL ontogeny may initiate from different BCS and that distinct mechanisms may lead to the emergence of cell clones with different immunogenetic properties (Agathangelidis et al. 2012). Indeed, the stereotyped BcR IG repertoire of CLL clones does not appear to reflect that of normal s-BCS and CD5^+^ B cells. On the other hand, the finding that CLL stereotyped BcR IG also can be expressed by MZ lymphoma clones, albeit rarely (Xochelli et al. 2018), suggests that B cell clones carrying certain BcR IG may give origin to different lymphoproliferations. These results point towards the notion that specific microenvironment signals (follicular versus extra-follicular) and/or by shared immune-mediated mechanisms (pathogen-driven) may be crucial for the ontogeny of lymphoid malignancies. In some instances, survival of the B cells expressing unmutated CBS-IG, particularly those shared by different individuals and thus belonging to the “public” BcR IG repertoire (Soto et al. 2019; Briney et al. 2019) could be warranted by particular types of antigen stimulation. This stimulation may not be leading to SHM or isotype switch and may be occur in the absence of a relevant T cell help or simply due to the fact their immunogenetic features are optimal for their role. The expression of CD5 by activated, transforming B cells (and the possible conversion to a stable expression of CD5 molecule by transformed cells) might be part of this stimulation process, possibly occurring during CLL leukemogenesis. However, several points remain unresolved, such as the relationship between the expression of CD5^+^ B cells identified in lymphoid tissues and those found in the circulation. These issues might be addressed in longitudinal studies, which are not, however, feasible on human tissues and requiring different experimental models.

## Conclusions

B cells with BcR IG rearrangements reminiscent of those belonging to the CLL major stereotyped subsets represent a sizeable cell fraction of the repertoire of different BCS from normal spleens and PB including CD5^+^ B cells that were significantly enriched compared to the CD5^−^ counterpart. However, the relative representation of CBS-IG was highly different from that of CLL in all B cell subgroups examined. Prime example was the rare identification of CLL subset #1, which is the most frequent observed in CLL. These stereotyped sequences appear to be part of the so-called “public” BcR IG repertoire (Soto et al. 2019; Briney et al. 2019). Altogether, the data presented here offer new insight into the mechanisms that are relevant to CLL ontogeny and suggest that, although potential CLL progenitors expressing stereotyped BcR IG are present in secondary lymphoid organs and in PB, additional mechanisms appear to be necessary for the generation of CLL clones.

## Supplementary information

**Additional file 1.**

**Additional file 2.**

## Data Availability

DNA sequences were deposited on Sequence Read Achive (SRA) portal of NCBI with BioProject ID: PRJNA515424.

## Abbreviations

BCS: B cell subpopulation
CBS-IG: CLL-biased stereotyped immunoglobulin
CLL: Chronic Lymphocytic Leukemia
DN: Double negative
FM: Follicular mantle
GC: Germinal center
MO: M-only
MZ: Marginal zone
s-BCS: Splenic B cell subpopulation
SM: Switched memory
SPL: Spleen
TR: Transitional
