# Supplementary material for: Tracing CLL-biased stereotyped immunoglobulin gene rearrangements in normal B cell subsets using a high-throughput immunogenetic approach
Source: Mol Med. 2020 Mar 10;26:25. doi: 10.1186/s10020-020-00151-9 (PMC7063734; doi:10.1186/s10020-020-00151-9)
Supplement: Supplementary file 1 — Additional file 1. [file 10020_2020_151_MOESM1_ESM.docx]

**Supplementary information**

**Tracing CLL-biased stereotyped immunoglobulin gene rearrangement in normal B cell subsets using a high-throughput immunogenetic approach**

Monica Colombo^1*^, Davide Bagnara^2*^, Daniele Reverberi^1^, Serena Matis^1^, Martina Cardillo^1^, Rosanna Massara^1^, Luca Mastracci^3,4^, Jean Louis Ravetti^3^, Luca Agnelli^5^, Antonino Neri^5^, Michela Mazzocco^6^, Margherita Squillario^7^, Andrea Nicola Mazzarello^8^, Giovanna Cutrona^1^, Andreas Agathangelidis^9^, Kostas Stamatopoulos^9^, Manlio Ferrarini^2^, Franco Fais^1,2^.

^1^U.O. Molecular Pathology, IRCCS Ospedale Policlinico San Martino, Genoa, Italy;

^2^Department of Experimental Medicine, University of Genoa, Genoa, Italy;

^3^U.O. Pathology, IRCCS Ospedale Policlinico San Martino, Genoa, Italy;

^4^Department of Surgical Sciences and Integrated Diagnostics, University of Genoa, Italy;

^5^Department of Oncology and Hemato-oncology, University of Milan, Milan, Italy;

^6^U.O. Laboratorio di Istocompatibilità, E.O. Ospedali Galliera, Genoa, Italy;

^7^Department of Informatic Bioengeneering, Robotic and System Engeneering, University of Genoa, Genoa, Italy;

^8^The Feinstein Institute for Medical Research, Manhasset, NY, USA;

^9^Institute of Applied Biosciences, Centre for Research and Technology Hellas, 6th km Harilaou-Thermi Road, Thessaloniki, Greece.

*The authors contributed equally to this work

Corresponding author: Monica Colombo, Molecular Pathology, IRCCS Ospedale Policlinico San Martino, Genoa, Italy, tel: +39 0105558972; e-mail: monica.colombo@hsanmartino.it

## Supplement Index:

Supplementary Materials and Methods pages 02-14

Supplementary Tables pages 15-17

Supplementary Figures pages 18-23

Supplementary Table S4, is provided separately as an excel-file

**Supplementary Material and methods**

1. Oligonucleotides specific for IGHV1 gene subgroup employed for Ig library preparation
2. Code 1 used for the processing of the raw reads
3. Code 2 used for the clonal assignment

**1**

| **NAME** | **ILLUMINA+RANDOM(14-16)+PRIMER FOR DS-CDNA** |
| --- | --- |
| **L3_VH1*2** | 5’-GCGTCAGATGTGTATAAGAGACAGNNNNNNNNNNNNNNCMACTACAGGWGCCCACTCC |
| **L3_VH1-46** | 5-’GCGTCAGATGTGTATAAGAGACAGNNNNNNNNNNNNNNTAGCTCCAGGTGCCCACTCC |
| **L3_VH1-69** | 5’-GCGTCAGATGTGTATAAGAGACAGGNNNNNNNNNNNNNNNNCAGCYACAGGTGTCCASTCC |
| **L3_VH1-2** | 5’-GCGTCAGATGTGTATAAGAGACAGGNNNNNNNNNNNNNNNCCACAGGWGCCCACTCC |
| **L3_VH1-45** | 5’-GCGTCAGATGTGTATAAGAGACAGNNNNNNNNNNNNNNAGCCACAGATGCCTACTCC |
| **L3_VH1-24** | 5’-GCGTCAGATGTGTATAAGAGACAGNNNNNNNNNNNNNNNCTACAGGCACCCACGCC |
|  | **PRIMERS FOR PCR** |
| **ILF1** | 5’-TCGTCGGCAGGCGTCAGATGTGTATAAGAGACAG |
| **HCM** | 5’-GTCTCGTGGGCTCGGAGATGTGTATAAGAGACAGAGTAGTCCTTGACCAGGCAGC |
| **HCG** | 5’-GTCTCGTGGGCTCGGAGATGTGTATAAGAGACAGAAAGGGTTGGGGCGGATGC |

**2.**

**Code 1: Sequencing data quality control The bash script used to run pRESTO.**

#!/bin/bash

#uper script to run the pRESTO 0.4.7 pipeline Dado's data

#

# Author: Jason Anthony Vander Heiden, Gur Yaari, Namita Gupta

# Date: 2015.05.31

#

# Required Arguments:

# $1 = read 1 file (V-region start sequence)

# $2 = read 2 file (C-region start sequence)

# $3 = output directory

# $4 = output file prefix

# $5 = number of subprocesses for multiprocessing tools

# Capture command line parameters

R1_FILE="$1" #(readlink -n $1)

R2_FILE="$2" #(readlink -n $2)

OUTDIR="$3"

OUTNAME="$4"

NPROC="$5"

# Define pipeline steps

ZIP_FILES=true

FILTER_LOWQUAL=true

ALIGN_UIDSETS=false

REFERENCE_ASSEMBLY=true

MASK_LOWQUAL=false

ALIGN_R2=false

OUTNAME_ID=true # attach to each seq name (BARCODE) the id of the sample

VAR_BARCODE=true # assume length of N, N-1 and N-2

# FilterSeq run parameters

FS_QUAL=20

FS_MASK=30

# MaskPrimers run parameters

R1_PRIMERS="/path/R1/primers.fasta"

R2_PRIMERS="/path/R2/primers.fasta"

MP_UIDLEN=16 #16 for variable UID put the longhest (N), assume N, N-1, N-2

MP_R1_MAXERR=0.2

MP_R2_MAXERR=0.3

MP_R1_MAXLEN=40

MP_R2_MAXLEN=100

MP_CREGION_MAXLEN=100

MP_CREGION_MAXERR=0.2 # 0.1

MP_CREGION_PRIMERS="/path/CREGION.fasta/" # constant region sequence in amplicon to identify isotype

# AlignSets run parameters

MUSCLE_EXEC=/usr/local/bin/muscle

# BuildConsensus run parameters

BC_PRCONS_FLAG=true

BC_ERR_FLAG=true

BC_MAXERR=0.2

BC_PRCONS=0.6

BC_QUAL=0

BC_MAXGAP=0.5

BC_MAXDIV=0.3

# AssemblePairs-align run parameters

AP_ALN_SCANREV=true

AP_ALN_MAXERR=0.3

AP_ALN_MINLEN=8

AP_ALN_ALPHA=1e-5

# AssemblePairs-reference run parameters

AP_REF_MINIDENT=0.5

AP_REF_EVALUE=1e-5

AP_REF_MAXHITS=100

REF_FILE="/path/V-germline/database.fasta" # IGHV germline ungapped

USEARCH_EXEC=/usr/local/bin/usearch

# CollapseSeq run parameters

CS_KEEP=false

CS_MISS=10

# Define log files

PIPELINE_LOG="Pipeline.log"

ERROR_LOG="Pipeline.err"

# Make output directory and empty log files

mkdir -p $OUTDIR; cd $OUTDIR

echo '' > $PIPELINE_LOG

echo '' > $ERROR_LOG

# Start

echo "DIRECTORY: ${OUTDIR}"

echo "VERSIONS:"

echo " $(AlignSets.py --version 2>&1)"

echo " $(AssemblePairs.py --version 2>&1)"

echo " $(BuildConsensus.py --version 2>&1)"

echo " $(ClusterSets.py --version 2>&1)"

echo " $(CollapseSeq.py --version 2>&1)"

echo " $(ConvertHeaders.py --version 2>&1)"

echo " $(FilterSeq.py --version 2>&1)"

echo " $(MaskPrimers.py --version 2>&1)"

echo " $(PairSeq.py --version 2>&1)"

echo " $(ParseHeaders.py --version 2>&1)"

echo " $(ParseLog.py --version 2>&1)"

echo " $(SplitSeq.py --version 2>&1)"

echo -e "\nSTART"

STEP=0

# Remove low quality reads

if $FILTER_LOWQUAL; then

printf " %2d: %-*s $(date +'%H:%M %D')\n" $((++STEP)) 24 "FilterSeq quality"

FilterSeq.py quality -s $R1_FILE -q $FS_QUAL --nproc $NPROC --outname "${OUTNAME}-R1" --outdir . --log QualityLogR1.log >> $PIPELINE_LOG 2> $ERROR_LOG

FilterSeq.py quality -s $R2_FILE -q $FS_QUAL --nproc $NPROC --outname "${OUTNAME}-R2" --outdir . --log QualityLogR2.log >> $PIPELINE_LOG 2> $ERROR_LOG

MPR1_FILE="${OUTNAME}-R1_quality-pass.fastq"

MPR2_FILE="${OUTNAME}-R2_quality-pass.fastq"

else

MPR1_FILE=$R1_FILE

MPR2_FILE=$R2_FILE

fi

# Add $OUTNAME to BARCODE field

if $OUTNAME_ID; then

printf " %2d: %-*s $(date +'%H:%M %D')\n" $((++STEP)) 24 "ParseHeaders add"

ParseHeaders.py add -s $MPR1_FILE -f BARCODE -u "${OUTNAME}_" --outname "${OUTNAME}-R1_sampleID" --outdir . >> $PIPELINE_LOG 2> $ERROR_LOG

MPR1_FILE="${OUTNAME}-R1_sampleID_reheader.fastq"

fi

printf " %2d: %-*s $(date +'%H:%M %D')\n" $((++STEP)) 24 "MaskPrimers score"

MaskPrimers.py score -s $MPR1_FILE -p $R1_PRIMERS --mode cut --failed --start $MP_UIDLEN --barcode --maxerror $MP_R1_MAXERR --nproc $NPROC --log PrimerLogR1.log --outname "${OUTNAME}-R1" --outdir . >> $PIPELINE_LOG 2> $ERROR_LOG

R1_PRIMER_PASS="${OUTNAME}-R1_primers-pass.fastq"

if $VAR_BARCODE; then

UIDLEN=$((MP_UIDLEN -1))

MaskPrimers.py score -s "${OUTNAME}-R1_primers-fail.fastq" -p $R1_PRIMERS --mode cut --failed --start $UIDLEN --barcode --maxerror $MP_R1_MAXERR --nproc $NPROC --log PrimerLogR1.log --outname "${OUTNAME}-R1-1" --outdir . >> $PIPELINE_LOG 2> $ERROR_LOG

UIDLEN=$((UIDLEN -1))

MaskPrimers.py score -s "${OUTNAME}-R1-1_primers-fail.fastq" -p $R1_PRIMERS --mode cut --failed --start $UIDLEN --barcode --maxerror $MP_R1_MAXERR --nproc $NPROC --log PrimerLogR1.log --outname "${OUTNAME}-R1-2" --outdir . >> $PIPELINE_LOG 2> $ERROR_LOG

cat "${OUTNAME}-R1_primers-pass.fastq" "${OUTNAME}-R1-1_primers-pass.fastq" "${OUTNAME}-R1-2_primers-pass.fastq" > "${OUTNAME}-R1-UID_primers-pass.fastq"

R1_PRIMER_PASS="${OUTNAME}-R1-UID_primers-pass.fastq"

else

R1_PRIMER_PASS="${OUTNAME}-R1_primers-pass.fastq"

fi

MaskPrimers.py score -s $MPR2_FILE -p $R2_PRIMERS --mode tag --start 0 --maxerror $MP_R2_MAXERR --nproc $NPROC --log PrimerLogR2.log --outname "${OUTNAME}-R2-m" --outdir . --failed >> $PIPELINE_LOG 2> $ERROR_LOG

# Collapse R2 PRIMER field

printf " %2d: %-*s $(date +'%H:%M %D')\n" $((++STEP)) 24 "ParseHeaders collapse"

ParseHeaders.py collapse -s "${OUTNAME}-R2-m_primers-pass.fastq" -f PRIMER --act first --outname "${OUTNAME}-R2-PR_collapse" --outdir . >> $PIPELINE_LOG 2> $ERROR_LOG

# Collapse BARCODE field

if $OUTNAME_ID; then

printf " %2d: %-*s $(date +'%H:%M %D')\n" $((++STEP)) 24 "ParseHeaders collapse"

ParseHeaders.py collapse -s $R1_PRIMER_PASS -f BARCODE --act cat --outname "${OUTNAME}-R1-ID_collapse" --outdir . >> $PIPELINE_LOG 2> $ERROR_LOG

MPR1_FILE="${OUTNAME}-R1-ID_collapse_reheader.fastq"

fi

# Assign UIDs to read 2 sequences

printf " %2d: %-*s $(date +'%H:%M %D')\n" $((++STEP)) 24 "PairSeq"

PairSeq.py -1 $MPR1_FILE -2 "${OUTNAME}-R2-PR_collapse_reheader.fastq" --outname "${OUTNAME}-R" --1f BARCODE --coord illumina >> $PIPELINE_LOG 2> $ERROR_LOG

# Multiple align UID read groups

if $ALIGN_UIDSETS; then

printf " %2d: %-*s $(date +'%H:%M %D')\n" $((++STEP)) 24 "AlignSets muscle"

AlignSets.py muscle -s "${OUTNAME}-R-1_pair-pass.fastq" --nproc $NPROC --log AlignLogR1.log --outname "${OUTNAME}-R1" >> $PIPELINE_LOG 2> $ERROR_LOG

AlignSets.py muscle -s "${OUTNAME}-R-2_pair-pass.fastq" --nproc $NPROC --log AlignLogR2.log --outname "${OUTNAME}-R2" >> $PIPELINE_LOG 2> $ERROR_LOG

BCR1_FILE="${OUTNAME}-R1_align-pass.fastq"

BCR2_FILE="${OUTNAME}-R2_align-pass.fastq"

else

BCR1_FILE="${OUTNAME}-R-1_pair-pass.fastq"

BCR2_FILE="${OUTNAME}-R-2_pair-pass.fastq"

fi

# Build UID consensus sequences

printf " %2d: %-*s $(date +'%H:%M %D')\n" $((++STEP)) 24 "BuildConsensus"

if $BC_ERR_FLAG; then

if $BC_PRCONS_FLAG; then

BuildConsensus.py -s $BCR2_FILE --bf BARCODE --pf PRIMER -q $BC_QUAL --maxerror $BC_MAXERR --nproc $NPROC --prcons $BC_PRCONS --log ConsensusLogR2.log --outname "${OUTNAME}-R2" >> $PIPELINE_LOG 2> $ERROR_LOG

else

BuildConsensus.py -s $BCR2_FILE --bf BARCODE --pf PRIMER -q $BC_QUAL --maxerror $BC_MAXERR --maxgap $BC_MAXGAP --nproc $NPROC --log ConsensusLogR2.log --outname "${OUTNAME}-R2" >> $PIPELINE_LOG 2> $ERROR_LOG

fi

BuildConsensus.py -s $BCR1_FILE --bf BARCODE --pf PRIMER -q $BC_QUAL --maxerror $BC_MAXERR --maxgap $BC_MAXGAP --nproc $NPROC --log ConsensusLogR1.log --outname "${OUTNAME}-R1" >> $PIPELINE_LOG 2> $ERROR_LOG

else

if $BC_PRCONS_FLAG; then

BuildConsensus.py -s $BCR2_FILE --bf BARCODE --pf PRIMER -q $BC_QUAL --maxgap $BC_MAXGAP --nproc $NPROC --prcons $BC_PRCONS--log ConsensusLogR2.log --outname "${OUTNAME}-R2" >> $PIPELINE_LOG 2> $ERROR_LOG

else

BuildConsensus.py -s $BCR2_FILE --bf BARCODE --pf PRIMER -q $BC_QUAL --maxgap $BC_MAXGAP --nproc $NPROC --log ConsensusLogR2.log --outname "${OUTNAME}-R2" >> $PIPELINE_LOG 2> $ERROR_LOG

fi

BuildConsensus.py -s $BCR1_FILE --bf BARCODE --pf PRIMER -q $BC_QUAL --maxgap $BC_MAXGAP --nproc $NPROC --log ConsensusLogR1.log --outname "${OUTNAME}-R1" >> $PIPELINE_LOG 2> $ERROR_LOG

fi

# Assign UIDs to read 2 sequences

printf " %2d: %-*s $(date +'%H:%M %D')\n" $((++STEP)) 24 "PairSeq"

PairSeq.py -1 "${OUTNAME}-R1_consensus-pass.fastq" -2 "${OUTNAME}-R2_consensus-pass.fastq" --coord presto >> $PIPELINE_LOG 2> $ERROR_LOG

# Assemble paired ends via mate-pair alignment

printf " %2d: %-*s $(date +'%H:%M %D')\n" $((++STEP)) 24 "AssemblePairs align"

if $BC_PRCONS_FLAG; then

PRFIELD="PRCONS"

else

PRFIELD="PRIMER"

fi

if $AP_ALN_SCANREV; then

AssemblePairs.py align -1 "${OUTNAME}-R1_consensus-pass_pair-pass.fastq" -2 "${OUTNAME}-R2_consensus-pass_pair-pass.fastq" --1f CONSCOUNT --2f $PRFIELD CONSCOUNT --coord presto --rc tail --minlen $AP_ALN_MINLEN --maxerror $AP_ALN_MAXERR --alpha $AP_ALN_ALPHA --nproc $NPROC --log AssembleAlignLog.log --outname "${OUTNAME}-ALN" --scanrev --failed >> $PIPELINE_LOG 2> $ERROR_LOG

else

AssemblePairs.py align -1 "${OUTNAME}-R1_consensus-pass_pair-pass.fastq" -2 "${OUTNAME}-R2_consensus-pass_pair-pass.fastq" --1f CONSCOUNT --2f $PRFIELD CONSCOUNT --coord presto --rc tail --minlen $AP_ALN_MINLEN --maxerror $AP_ALN_MAXERR --alpha $AP_ALN_ALPHA --nproc $NPROC --log AssembleAlignLog.log --outname "${OUTNAME}-ALN" --failed >> $PIPELINE_LOG 2> $ERROR_LOG

fi

# Assemble paired ends via alignment against V-region reference database

if $REFERENCE_ASSEMBLY; then

printf " %2d: %-*s $(date +'%H:%M %D')\n" $((++STEP)) 24 "AssemblePairs reference"

AssemblePairs.py reference -1 "${OUTNAME}-ALN-1_assemble-fail.fastq" -2 "${OUTNAME}-ALN-2_assemble-fail.fastq" -r $REF_FILE --1f CONSCOUNT --2f $PRFIELD CONSCOUNT --coord presto --minident $AP_REF_MINIDENT --evalue $AP_REF_EVALUE --maxhits $AP_REF_MAXHITS --nproc $NPROC --log AssembleReferenceLog.log --outname "${OUTNAME}-REF" --failed >> $PIPELINE_LOG 2> $ERROR_LOG

cat "${OUTNAME}-ALN_assemble-pass.fastq" "${OUTNAME}-REF_assemble-pass.fastq" > "${OUTNAME}-CAT_assemble-pass.fastq"

PH_FILE="${OUTNAME}-CAT_assemble-pass.fastq"

else

PH_FILE="${OUTNAME}-ALN_assemble-pass.fastq"

fi

# Mask low quality positions

if $MASK_LOWQUAL; then

printf " %2d: %-*s $(date +'%H:%M %D')\n" $((++STEP)) 24 "FilterSeq maskqual"

FilterSeq.py maskqual -s $PH_FILE -q $FS_MASK --nproc $NPROC --outname "${OUTNAME}-MQ" --log MaskqualLog.log >> $PIPELINE_LOG 2> $ERROR_LOG PH_FILE="${OUTNAME}-MQ_maskqual-pass.fastq"

fi

# Rewrite header with minimum of CONSCOUNT

printf " %2d: %-*s $(date +'%H:%M %D')\n" $((++STEP)) 24 "ParseHeaders collapse"

ParseHeaders.py collapse -s $PH_FILE -f CONSCOUNT --act min --outname "${OUTNAME}-FIN" > /dev/null 2> $ERROR_LOG

#####################

# detect CREGION with MaskPrimers.py align

# Annotate with internal C-region

printf " %2d: %-*s $(date +'%H:%M %D')\n" $((++STEP)) 24 "MaskPrimers score"

MaskPrimers.py score -s "${OUTNAME}-FIN_reheader.fastq" -p $MP_CREGION_PRIMERS --maxerror $MP_CREGION_MAXERR --mode cut --revpr --start 0 --failed --log CRegionLog.log --outname "${OUTNAME}-CR" --nproc $NPROC >> $PIPELINE_LOG 2> $ERROR_LOG

# Collapse CR PRIMER field

printf " %2d: %-*s $(date +'%H:%M %D')\n" $((++STEP)) 24 "ParseHeaders collapse"

ParseHeaders.py collapse -s "${OUTNAME}-CR_primers-pass.fastq" -f PRIMER --act first --outname "${OUTNAME}-CR-primer_collapse" --outdir . >> $PIPELINE_LOG 2> $ERROR_LOG

# Renamer primer field

printf " %2d: %-*s $(date +'%H:%M %D')\n" $((++STEP)) 24 "ParseHeaders rename"

ParseHeaders.py rename -s "${OUTNAME}-CR-primer_collapse_reheader.fastq" -f PRIMER -k CREGION --outname "${OUTNAME}-CR" > /dev/null 2> $ERROR_LOG

#################

# Remove duplicate sequences

printf " %2d: %-*s $(date +'%H:%M %D')\n" $((++STEP)) 24 "CollapseSeq"

if $CS_KEEP; then

CollapseSeq.py -s "${OUTNAME}-CR_reheader.fastq" -n $CS_MISS --uf PRCONS --cf CONSCOUNT CREGION --act sum set --inner --fasta --keepmiss --outname "${OUTNAME}-FIN" >> $PIPELINE_LOG 2> $ERROR_LOG

else

CollapseSeq.py -s "${OUTNAME}-CR_reheader.fastq" -n $CS_MISS --uf PRCONS --cf CONSCOUNT CREGION --act sum set --inner --fasta --outname "${OUTNAME}-FIN" >> $PIPELINE_LOG 2> $ERROR_LOG

fi

###############################

# Filter to sequences with at least 2 supporting sources

printf " %2d: %-*s $(date +'%H:%M %D')\n" $((++STEP)) 24 "SplitSeq group"

SplitSeq.py group -s "${OUTNAME}-FIN_collapse-unique.fasta" -f CONSCOUNT --num 2 --fasta >> $PIPELINE_LOG 2> $ERROR_LOG

# Create table of final repertoire

printf " %2d: %-*s $(date +'%H:%M %D')\n" $((++STEP)) 24 "ParseHeaders table"

ParseHeaders.py table -s "${OUTNAME}-FIN_collapse-unique.fasta" -f ID PRCONS CREGION CONSCOUNT DUPCOUNT --outname "${OUTNAME}-Final-Unique" >> $PIPELINE_LOG 2> $ERROR_LOG

ParseHeaders.py table -s "${OUTNAME}-FIN_collapse-unique_atleast-2.fasta" -f ID PRCONS CREGION CONSCOUNT DUPCOUNT --outname "${OUTNAME}-Final-Unique-Atleast2" >> $PIPELINE_LOG 2> $ERROR_LOG

# Process log files

printf " %2d: %-*s $(date +'%H:%M %D')\n" $((++STEP)) 24 "ParseLog"

if $FILTER_LOWQUAL; then

ParseLog.py -l QualityLogR[1-2].log -f ID QUALITY > /dev/null &

fi

ParseLog.py -l PrimerLogR[1-2].log -f ID BARCODE PRIMER ERROR > /dev/null 2> $ERROR_LOG &

ParseLog.py -l ConsensusLogR[1-2].log -f BARCODE SEQCOUNT CONSCOUNT PRIMER PRCONS PRCOUNT PRFREQ ERROR > /dev/null 2> $ERROR_LOG &

ParseLog.py -l AssembleAlignLog.log -f ID LENGTH OVERLAP ERROR PVALUE FIELDS1 FIELDS2 > /dev/null 2> $ERROR_LOG &

if $REFERENCE_ASSEMBLY; then

ParseLog.py -l AssembleReferenceLog.log -f ID REFID LENGTH OVERLAP GAP EVALUE1 EVALUE2 IDENTITY FIELDS1 FIELDS2 > /dev/null 2> $ERROR_LOG &

fi

if $MASK_LOWQUAL; then

ParseLog.py -l MaskqualLog.log -f ID MASKED > /dev/null 2> $ERROR_LOG &

fi

if $ALIGN_CREGION; then

ParseLog.py -l CRegionLog.log -f ID PRIMER ERROR > /dev/null 2> $ERROR_LOG &

fi

wait

printf "DONE\n\n"

cd ../

**3.**

**Code 2**

**Clustering sequences in clonal groups**

#Automated threshold determination via smoothed density

output <- findThreshold(dist_ham$DIST_NEAREST, method="density")

threshold <- output@threshold

#Assigning Clones

DefineClones.py –d (File.db) –act set –model ham –norm len –dist (as above calculated)

**Supplementary Tables**

| s-BCS | Sorting mask used |
| --- | --- |
| Follicolar Mantle  FM | CD19+IgD++ CD38- IgM++CD27- |
| Germinal Center  GC | CD19+IgD-CD38+CD24- |
| Marginal Zone  MZ | CD19+IgDlow CD38- IgM++ |
| Switched memory  SM | CD19+IgD-CD38-CD27+IgM- |
| Double negative  DN | CD19+IgD-CD38-CD27-IgM- |
| IgM-only memory  MO | CD19+IgDneg CD38-IgM++ |
| Transitional  TR | CD19+CD24++CD38++ |
| (Naive)Marginal zone  MZ27- | CD19+IgDlow CD38-IgM++ CD27- |
| Memory Marginal zone  MZ27+ | CD19+IgDlow CD38-IgM++CD27+ |

**Supplementary Table 1**

**Mask sorting profile of splenic B cell subpopulations (s-BCS) separated by FACS**

**Supplementary Table 2**

**Main features of single cell-subpopulations included in the study**

| **Sample** | | **N. sorted cells** | | **Nr. reads** | | **Unique IGHV1_IGHD_IGHJ** | | **Threshold** | | **Nr. IGHV1 CLONES** | | **Nr. CBS-IG** | |
| --- | --- | --- | --- | --- | --- | --- | --- | --- | --- | --- | --- | --- | --- |
| **SPL19** | |  | |  | | **6519** | |  | | **4892** | | **19** | |
| DN | | 20000 | | 262191 | | 841 | | 0.2 | | 692 | | 0 | |
| FM | | 20000 | | 394952 | | 2111 | | 0.16 | | 1670 | | 15 | |
| MO | | 20000 | | 440337 | | 539 | | 0.14 | | 318 | | 0 | |
| MZ27- | | 20000 | | 363655 | | 555 | | 0.16 | | 450 | | 0 | |
| MZ27+ | | 20000 | | 278648 | | 567 | | 0.17 | | 370 | | 0 | |
| SM | | 20000 | | 462176 | | 736 | | 0.2 | | 535 | | 0 | |
| TR | | 20000 | | 975845 | | 1170 | | 0.075 | | 857 | | 4 | |
| **SPL25** | |  | |  | | **49999** | |  | | **26301** | | **65** | |
| FM | | 125000 | | 751531 | | 25173 | | 0.04 | | 19869 | | 50 | |
| GC | | 25000 | | 226824 | | 5817 | | 0.04 | | 618 | | 1 | |
| MZ | | 25000 | | 597123 | | 9459 | | 0.04 | | 2262 | | 12 | |
| SM | | 50000 | | 1024895 | | 9550 | | 0.06 | | 3552 | | 2 | |
| **SPL26** | |  | |  | | **33187** | |  | | **22649** | | **43** | |
| DN | | 30000 | | 213554 | | 2660 | | 0.04 | | 1656 | | 1 | |
| FM | | 30000 | | 305473 | | 4382 | | 0.04 | | 3736 | | 6 | |
| GC | | 30000 | | 318252 | | 2275 | | 0.09 | | 574 | | 0 | |
| MO | | 30000 | | 297871 | | 2681 | | 0.08 | | 1435 | | 1 | |
| MZ | | 30000 | | 283078 | | 5735 | | 0.08 | | 4401 | | 8 | |
| MZ27- | | 30000 | | 406112 | | 7784 | | 0.07 | | 5545 | | 18 | |
| MZ27+ | | 30000 | | 278680 | | 3191 | | 0.08 | | 2212 | | 2 | |
| SM | | 30000 | | 1015351 | | 2335 | | 0.05 | | 1673 | | 2 | |
| TR | | 30000 | | 306283 | | 2144 | | 0.03 | | 1417 | | 5 | |
| **SPL32** | |  | |  | | **9026** | |  | | **6786** | | **25** | |
| DN | | 20000 | | 577157 | | 766 | | 0.18 | | 635 | | 0 | |
| FM | | 40000 | | 1185639 | | 4390 | | 0.08 | | 3304 | | 14 | |
| GC | | 30000 | | 855562 | | 1256 | | 0.14 | | 633 | | 0 | |
| MO | | 20000 | | 384182 | | 391 | | 0.18 | | 247 | | 0 | |
| MZ27- | | 20000 | | 497860 | | 442 | | 0.11 | | 301 | | 1 | |
| MZ27+ | | 20000 | | 277071 | | 549 | | 0.16 | | 350 | | 0 | |
| SM | | 20000 | | 487388 | | 539 | | 0.2 | | 412 | | 1 | |
| TR | | 20000 | | 489667 | | 1241 | | 0.08 | | 904 | | 9 | |
| **SPL41** | |  | |  | | **10811** | |  | | **10811** | | 28 | |
| DN | | 20000 | | 678231 | | 1309 | | 0.2 | | 1003 | | 1 | |
| FM | | 40000 | | 1257991 | | 5770 | | 0.09 | | 4320 | | 13 | |
| MO | | 20000 | | 496303 | | 948 | | 0.16 | | 623 | | 0 | |
| MZ27- | | 20000 | |  | | 3230 | | 0,26 | | 2308 | | 6 | |
| MZ27+ | | 20000 | | 850246 | | 1483 | | 0.11 | | 876 | | 1 | |
| SM | | 20000 | | 592661 | | 1056 | | 0.2 | | 724 | | 0 | |
| TR | | 20000 | | 371244 | | 1271 | | 0.08 | | 957 | | 7 | |
| **SPL713** | |  | |  | | **73241** | |  | | **45588** | | **99** | |
| DN | | 31000 | | 366941 | | 4195 | | 0.08 | | 2946 | | 4 | |
| FM | | 30000 | | 646554 | | 12571 | | 0.04 | | 7264 | | 19 | |
| GC | | 42000 | | 693637 | | 6386 | | 0.04 | | 2216 | | 5 | |
| MO | | 28000 | | 645223 | | 6358 | | 0.08 | | 1859 | | 4 | |
| MZ | | 65000 | | 281426 | | 10499 | | 0.1 | | 8956 | | 21 | |
| MZ27- | | 58000 | | 473124 | | 13083 | | 0.04 | | 10629 | | 24 | |
| MZ27+ | | 43000 | | 61361 | | 2583 | | 0.1 | | 2040 | | 3 | |
| SM | | 35000 | | 338296 | | 4285 | | 0.04 | | 2417 | | 3 | |
| TR | | 29000 | | 1004078 | | 13281 | | 0.05 | | 7261 | | 16 | |
| **CD5+ and CD5- B CELLS** | | | |  | |  | |  | |  | |  | |
|  | |  | |  | |  | |  | |  | |  | |
| **SPL19** | |  | |  | |  | |  | |  | |  | |
| CD5+ | | 40000 | | 1388167 | | 1586 | | 0.070 | | 1586 | | 10 | |
| CD5- | | 80000 | | 1282105 | | 2632 | | 0.070 | | 2596 | | 4 | |
| **SPL32** | |  | |  | |  | |  | |  | |  | |
| CD5+ | | 20000 | | 554475 | | 1223 | | 0.070 | | 1223 | | 6 | |
| CD5- | | 20000 | | 485514 | | 820 | | 0.070 | | 820 | | 1 | |
| **SPL41** | |  | |  | |  | |  | |  | |  | |
| CD5+ | | 20000 | | 488750 | | 1720 | | 0.070 | | 1720 | | 10 | |
| CD5- | | 20000 | | 666365 | | 1136 | | 0.070 | | 1136 | | 4 | |
| **PB-AZ** | |  | |  | |  | |  | |  | |  | |
| CD5+ | | 10000 | | 109953 | | 1379 | | 0.070 | | 1283 | | 10 | |
| CD5- | | 10000 | | 123394 | | 768 | | 0.070 | | 709 | | 1 | |
| **PB-EC** | |  | |  | |  | |  | |  | |  | |
| CD5+ | | 10000 | | 158832 | | 910 | | 0.070 | | 835 | | 0 | |
| CD5- | | 10000 | | 164499 | | 835 | | 0.070 | | 767 | | 1 | |
| **PB-WS** | |  | |  | |  | |  | |  | |  | |
| CD5+ | | 10000 | | 109811 | | 1565 | | 0.070 | | 1465 | | 14 | |
| CD5- | | 10000 | | 155299 | | 904 | | 0.070 | | 838 | | 1 | |

**Supplementary Table 3: Major features Clan I CLL subsets^a^ using IGHV1 genes**

| CLL subset^a^ | #Cases | IGHV1 genes characterizing the subset | IGHJ genes | CDR3 length | Mutational status |
| --- | --- | --- | --- | --- | --- |
| #1 | 151 | IGHV1-2 IGHV1-3 | IGHJ4 | **13** | **UM** |
| #3 | 42 | IGHV1-69 | IGHJ6 | **22** | **UM** |
| #5 | 51 | IGHV1-69 | IGHJ6 | **20** | **UM** |
| #6 | 68 | IGHV1-69 | IGHJ6 | **21** | **UM** |
| #7H | 23 | IGHV1-69 | IGHJ6 | **24** | **UM** |
| #12 | 21 | IGHV1-2 IGHV1-46 | IGHJ4 | **19** | **UM** |
| #28A | 21 | IGHV1-2 IGHV1-3 | IGHJ6 | **17** | **UM** |
| #99 | 18 | IGHV1-2 IGHV1-3 | IGHJ4 | **14** | **UM** |
| #59 | 22 | IGHV1-69 IGHV1-58 | IGHJ4  IGHJ5 | **12** | **UM** |
|  |  |  |  |  |  |

^a^ CLL set reported were from Agathangelidis A, Darzentas N, Hadzidimitriou A, Brochet X, Murray F, Yan XJ, et al. Stereotyped B-cell receptors in one-third of chronic lymphocytic leukemia: a molecular classification with implications for targeted therapies. Blood. 2012;119(19):4467-75 and revised in “Encyclopedia of CLL subsets” at: http://station5.arrest.tools/subsets/.

**Supplementary Figures**

**Supplementary Figure 1.** Representation of FACS sorting mask **A** CD19+ B cells were gated according to the expression of CD38 and IgD (top line, center) and further separated into different B cell subpopulations. Phenotype profile of Follicular Mantle (FM) (top line, right), Germinal center (GC) (top-line left), Switched-Memory (SM) B cells (middle line, center), double-negative (DN) B cells (middle line, left), IgM only (MO) and Marginal Zone (MZ) B cells (middle line, center) are reported in supplementary Table 1. The MZ B cells were in turn fractionated into CD27+ (MZ27+) and CD27- (MZ27-) B cells (middle line, right). **B**. Purification of Transitional (TR) B cells from gated CD19+ B cells. TR B cells represented the CD38++, CD24++ cell fraction as indicated. Dot plots refer to the FACS sorting mask utilized for SPL713 purification.


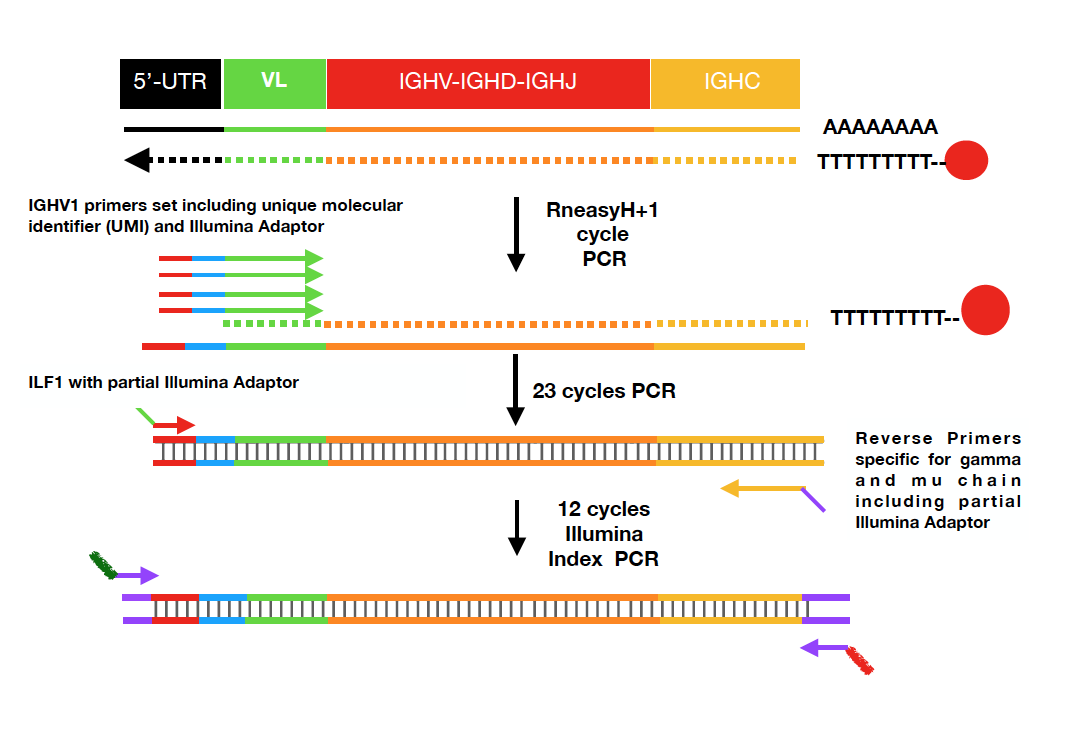


**Supplementary Figure 2**. Graphic representation of the strategy used for library preparation**.** RNA from purified B cell subpopulations was bound to oligo-dT-beads and retro-transcribed on the beads. IGH specific double strand cDNA with IGHV1 leader specific multiplex primers was made on the beads-bound cDNA after RNase H treatment. The above steps were followed by 23 PCR cycle for the amplification of the IGH with gamma and mu constant specific primers and a final 12 cycle Illumina PCR indexing.

**Supplementary Figure 3.** Distribution of stereotype subsets utilizing IGHV1 family genes among CBS-IG rearrangements of splenic B cells (s-BCS). This distribution is compared to that observed in a cohort of CLL cases reported in {Agathangelidis, 2012 #20} and summarized in supplementary Table 3. Only the CLL cases utilizing IGHV1 family genes were considered for this comparison. The relative frequencies of stereotype subsets in CLL and CBS-IG rearrangements from s-BCS are significantly different (p=0.00049).

**Supplementary Figure 4**. **A** Distribution of stereotype subsets among typical CBS-IG and non-typical CBS-IG rearrangements in splenic B cells subpopulations. The frequency distribution between typical and non-typical CBS-IG rearrangements in B cells considered in bulk was statistically different (p=0.001). **B** Frequency distribution of typical and non-typical CBS-IG rearrangements observed in the various relative B cell subpopulations as indicated at the bottom of the figure.

**Supplementary Figure 5**

Comparison of the relative CLL stereotyped subset frequencies detected in the total splenic B cells (A), in the CD5+ B cells from PB and spleens (B) and in the patient CLL cohort investigated by Agathangelidis et al. (C) using IGHV1 genes. Spleen numbers and PB donor initials are shown. Notably, donor EC did not exhibit CSB-IG rearrangements.


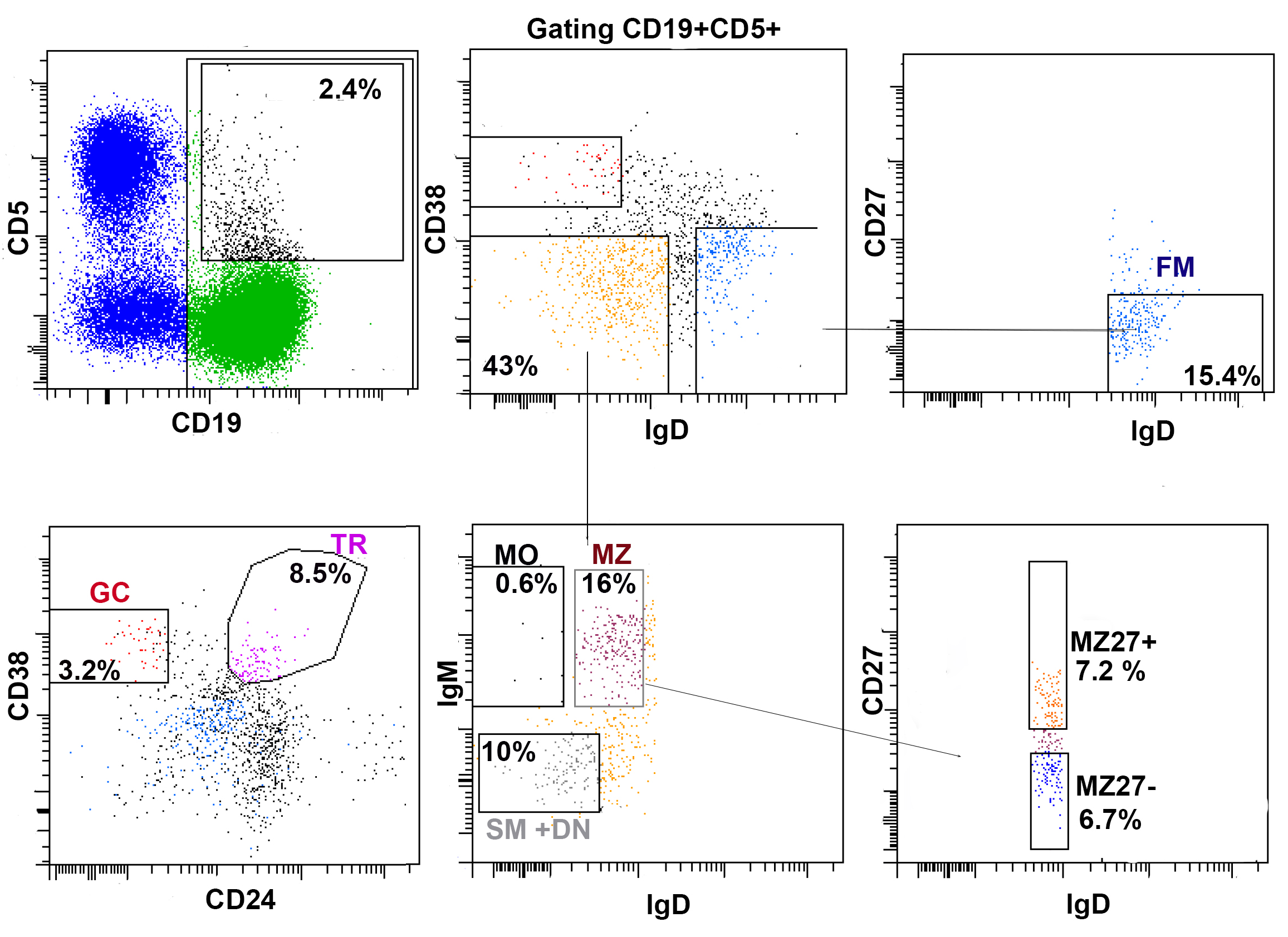


**Supplementary Figure 6**

Localization of CD5^+^ B cells within the different s-BCS. A representative experiment on SPL19 is shown. In this spleen CD5^+^ B cells were the 2.4% of the total B cells (top left panel). CD5^+^ B cells were the 4.2% and 7.3% among total B cells from spleen 32 and form spleen 41, respectively. FM (range 15.4-26.0%) and MZ (range 11.2-16.0%) were the most numerous subpopulations represented in the CD5^+^ B cells in the three spleens (right panels of the top and bottom center panel, respectively). The switched memory B cells represented from 4.0 to 10.0% of the CD5^+^ B cells (bottom center panel), TR represented from 8.5 to 12.5% of the CD5^+^ B cells and GC from 1.6-3.2% of the CD5^+^ B cells (bottom left panel). Finally, less of 1% (range 0.6-0.9%) of CD5^+^ B cells were represented in MO B cells (bottom center panel).
